# The effect of varying multidrug-resistence (MDR) definitions on rates of MDR gram-negative rods

**DOI:** 10.1186/s13756-019-0614-3

**Published:** 2019-11-28

**Authors:** Aline Wolfensberger, Stefan P. Kuster, Martina Marchesi, Reinhard Zbinden, Michael Hombach

**Affiliations:** 1 Division of Infectious Diseases and Hospital Epidemiology, University Hospital and University of Zurich, Rämistrasse 100, CH-8091 Zurich, Switzerland; 2 Institute of Medical Microbiology, University of Zurich, Zurich, Switzerland; 3 Present address: Roche Diagnostics International AG, Rotkreuz, Switzerland

**Keywords:** MDRO, gram-negatives, Multidrug-resistance, ECDC, KRINKO

## Abstract

**Background:**

A multitude of definitions determining multidrug resistance (MDR) of Gram-negative organisms exist worldwide. The definitions differ depending on their purpose and on the issueing country or organization. The MDR definitions of the European Centre for Disease Prevention and Control (ECDC) were primarily chosen to harmonize epidemiological surveillance. The German Commission of Hospital Hygiene and Infection Prevention (KRINKO) issued a national guideline which is mainly used to guide infection prevention and control (IPC) measures. The Swiss University Hospital Zurich (UHZ) – in absentia of national guidelines – developed its own definition for IPC purposes. In this study we aimed to determine the effects of different definitions of multidrug-resistance on rates of Gram-negative multidrug-resistant organisms (GN-MDRO).

**Methods:**

MDR definitions of the ECDC, the German KRINKO and the Swiss University Hospital Zurich were applied on a dataset comprising isolates of *Escherichia coli*, *Klebsiella pneumoniae, Enterobacter* sp.*, Pseudomonas aeruginosa,* and *Acinetobacter baumannii* complex. Rates of GN-MDRO were compared and the percentage of patients with a GN-MDRO was calculated.

**Results:**

In total 11′407 isolates from a 35 month period were included. For *Enterobacterales* and *P. aeruginosa,* highest MDR-rates resulted from applying the ‘ECDC-MDR’ definition. ‘ECDC-MDR’ rates were up to four times higher compared to ‘KRINKO-3/4MRGN’ rates, and up to six times higher compared to UHZ rates. Lowest rates were observed when applying the ‘KRINKO-4MRGN’ definitions. Comparing the ‘KRINKO-3/4MRGN’ with the UHZ definitions did not show uniform trends, but yielded higher rates for *E. coli* and lower rates for *P. aeruginosa*. On the patient level, the percentages of GN-MDRO carriers were 2.1, 5.5, 6.6, and 18.2% when applying the ‘KRINKO-4MRGN’, ‘UHZ-MDR’, ‘KRINKO-3/4MRGN’, and the ‘ECDC-MDR’ definition, respectively.

**Conclusions:**

Different MDR-definitions lead to considerable variation in rates of GN-MDRO. Differences arise from the number of antibiotic categories required to be resistant, the categories and drugs considered relevant, and the antibiotic panel tested. MDR definitions should be chosen carefully depending on their purpose and local resistance rates, as definitions guiding isolation precautions have direct effects on costs and patient care.

## Background

The number of gram-negative bacteria that are resistant to multiple antibiotics is on a constant rise and infections due to these resistant organisms pose an increasing threat to the achievements of modern medicine [1, 2]. Besides standard infection prevention precautions, most hospitals apply additional transmission-based precautions to reduce the spread of gram-negative multidrug-resistant organisms (GN-MDRO) from colonized or infected patients to others. Whether or not care for a patient with a GN-MDRO needs additional precautions deserves judgement about the organism’s clinical and epidemiological significance [3], as isolation precautions cause high direct and indirect costs, and were shown to negatively impact several dimensions of patient care [4, 5]. It is well known that the definitions of multidrug resistance (MDR) are neither harmonized between countries, nor between hospitals in the same country, nor do guidelines on infection prevention and control (IPC) standards for patients with GN-MDRO exist to date [6–8].

In Europe, a multitude of definitions for GN-MDRO with varying purposes do exist: The MDR definitions of the European Centre for Disease Prevention and Control (ECDC), published in 2011, were primarily chosen to harmonize epidemiological surveillance data across healthcare settings and countries [9]. The ECDC criteria define MDR as acquired non-susceptibility to at least one agent in three or more antimicrobial categories. Categories were constructed with the intent of placing antimicrobial agents into therapeutically relevant groups and each category is considered equally relevant.

Germany has a nationwide MDR definition, issued by the national German Commission of Hospital Hygiene and Infection Prevention (KRINKO) in 2012, which is primarily used to guide IPC measures [10]. The German KRINKO defines MDR according to resistance to commonly used agents to treat severe infections (i.e. antipseudomonal penicillins, extended spectrum cephalosporins, carbapenems and quinolones). It grades resistance by severity into multidrug resistance to three and four antibiotic categories. Isolation precautions are recommended for all patients with bacterial species resistant to four antibiotic categories. For patients with species resistant to three antibiotic categories, isolation precautions are warranted for patients on high-risk wards (e.g. hemato-oncology) with *Escherichia coli, Klebsiella* sp*., Enterobacter* sp*., Pseudomonas aeruginosa,* and *Acinetobacter baumanii*.

In Switzerland, no national consensus guidelines on MDR definitions exist to date. Therefore, the University Hospital Zurich (UHZ) developed its own definition in 2008. These definitions are based on clinical reasoning, local resistance rates, antibiotic use policies, and international expert proposals [11]. They are mainly used to guide IPC measures. The UHZ-guidelines define MDR as resistance to agents of three out of five antimicrobial categories including the aminoglycosides. Unlike the ECDC and KRINKO definitions, the UHZ also includes the ESBL-phenotype of *Enterobacterales* (except *Escherichia coli*) as MDR. *E. coli* ESBL is not considered a GN-MDRO following recommendations of the Swiss national center for infection control (Swissnoso) recommending standard precautions for this specific ESBL-producing species [12].

Differences in MDR definitions do have an impact on percentage of gram-negatives considered MDRO and, if used to guide IPC measures, on patients requiring isolation precautions. The primary aim of this study was to analyze the impact of the ECDC, KRINKO and UHZ definitions on the rate of GN-MDRO and the resulting number of patients characterized as GN-MDRO carriers. To describe the influence of the different MDR definition criteria, we applied the definition criteria of ECDC, KRINKO and UHZ on 11′407 isolates of five common gram-negative species isolated during a 35 month period in patients from the UHZ.

## Methods

### Setting

The University Hospital Zurich, Zurich, Switzerland, is an 950-bed tertiary-care teaching hospital covering all medical specialties except paediatrics and orthopaedics. All microbiologic samples are tested in the clinical microbiology laboratory of the Institute of Medical Microbiology, University of Zurich, Zurich, Switzerland. The Institute of Medical Microbiology collects all raw data of disk diffusion antimicrobial susceptibility testing (i.e. inhibition zone diameters) in a dedicated database (Sirweb, i2a, Montpellier, France), allowing re-analysis of the raw data.

### Isolates and patients

Data from gram-negative rods isolated during a 35-month period from 1.1.2013 to 1.12.2015 were analysed. Species analysed comprised *Escherichia coli*, *Klebsiella pneumoniae, Enterobacter* sp., *Pseudomonas aeruginosa,* and *Acinetobacter baumannii* complex. In order to prevent the exclusion of potential MDR follow-up isolates which arise from selective pressure under therapy, we intentionally included all repeat isolates, neglecting guidelines for analysis and presentation of cumulative antibiograms advising inclusion of first isolates per patient only [13]. We only included isolates with a miminum of the following antibiotics tested (required for the ‘UHZ-2008’ definitions, see below): piperacillin/tazobactam, ceftriaxone (for *Enterobacterales*), ceftazidime, cefepime, ertapenem (for *Enterobacterales*), imipenem, meropenem, levofloxacin, ciprofloxacin, amikacin, tobramycin and gentamicin. In total, we included 11.407 isolates from 8.454 patients.

To assess the percentage of patients with colonisation or infection with a GN-MDRO, we divided the number of patients with at least one GN-MDRO by the total patient population included in the study.

### Susceptibility testing, detection of resistance mechanisms and species identification

Only data collected during routine diagnostic testing was included in this study. For susceptibility testing, the disc diffusion method according to Kirby-Bauer was used. Antibiotic discs were obtained from i2a (Montpellier, France). Susceptibility testing was done on Mueller-Hinton agar (Becton-Dickinson, Franklin Lakes, NJ, USA) using MacFarland 0.5 from overnight cultures followed by incubation at 35 °C for 16-18 h. Inhibition zone diameters were determined and recorded in the automated Sirweb/Sirscan system (i2a) and interpreted according to EUCAST 2015 guidelines [14]. When EUCAST provided no interpretation guidelines for the tested antibiotics, CLSI 2015 guidelines were applied [15]: ceftriaxone, cefepime, ceftazidime and piperacillin/tazobactam for *A. baumannii* complex, fosfomycin for the *Enterobacterales*, minocycline and tetracycline for *Enterobacterales* and *A. baumannii* complex. Since July 2015 susceptibility to ciprofloxacin and levofloxacin of *Enterobacterales* was inferred from the susceptibility from norfloxacin. Intermediate susceptibility is interpreted as resistant.

ESBL production was phenotypically detected by using screening cut-off values for cephalosporines, followed by phenotypic confirmation with combination-disk and double-disk synergy tests and, in non-distinctive cases, molecular detection of ESBL genes [16, 17]. Carbapenemase production was phenotypically detected by using screening cut-off values for meropenem, followed by phenotypic confirmation with combination disk tests and molecular detection of carbepenemase genes [16, 18, 19].

Species identification was performed by matrix-assisted laser desorption ionization-time of flight mass spectrometry (MALDI-TOF MS) by the direct transfer-formic acid method using Bruker Biotyper MALDI-TOF MS System (Bruker corporation) [20].

### MDR-definitions of ECDC, KRINKO and UHZ

The three different MDR definitions per bacterial species are depicted in Tables 1, 2 and 3.

**Table 1:** Definition criteria and antibiotic panel for *Enterobacterales* (*Escherichia coli*, *Klebsiella pneumoniae* and *Enterobacter* sp.)

Antimicrobial category	ECDC-MDR	KRINKO-3MRGN	KRINKO-4MRGN	UHZ-MDR
Antipseudomonal penicillins	Piperacillin/ tazobactam or *ticarcillin/ clavulanic acid*	*Piperacillin* ^a)^	*Piperacillin* ^a)^	Piperacillin/ tazobactam
Extended spectrum cephalosporins	*Cefotaxime* or ceftriaxone or ceftazidime or cefepime	*Cefotaxime* ^b)^ or ceftazidime	*Cefotaxime* ^b)^ or ceftazidime	Ceftriaxone and ceftazidime and cefepime
Carbapenems	Ertapenem or imipenem or meropenem or *doripenem*	Imipenem or meropenem	Imipenem or meropenem	≥ 2 of: Ertapenem, imipenem, meropenem
Quinolones	Ciprofloxacin	Ciprofloxacin	Ciprofloxacin	Ciprofloxacin and levofloxacin
Aminoglycosides	Amikacin or gentamicin or tobramycin or *netilmicin*			≥2 of: Amikacin, gentamicin, tobramycin
Monobactams	*Aztreonam*			
Phosphonic acids	Fosfomycin			
non-extended spectrum cepholosporins	*Cefazolin* ^c)^ or cefuroxime			
anti-MRSA cephalosporins ^e)^	*Ceftaroline*			
Cephamycins	*Cefoxitin* ^c)^ or *Cefotetan* ^c)^			
Folate pathway inhibitors	Trimethoprim/ sulphamethoxazol			
Glycylcyclines	Tigecycline			
Penicillins	Ampicillin ^c) d)^			
Penicillins plus beta-lactamase-inhibitors	Amoxicillin/ clavulanic acid ^c)^ or *ampicillin/ sulbactam* ^c)^			
Phenicols	*Chloramphenicol*			
Polymyxins	*Colistin or polymyxin B*			
Tetracyclines	Tetracycline or *doxycycline* or minocycline			
Definition of MDR	Resistant to ≥3 categories	Resistant to antipseudomonal penicillin and cephalosporin-group and quinolone and susceptible to carbapenems	Resistant to all 4 categories or if a carbapenem is resistant all Enterobacterales are interpreted as 4MRGN or Enterobacterales with carbapenemase production	Resistant to 3 out of 5 categories or ESBL-producing Enterobacterales (except E.coli) or Enterobacterales with carbapenemase production

Abbreviations: *ECDC-MDR* Multidrug resistance according to the European Centre for Disease Prevention and Control, *KRINKO-3MRGN* Multidrug resistance to three antibiotic categories according to the German Commission of Hospital Hygiene and Infection Prevention, *KRINKO-4MRGN* Multidrug resistance to four antibiotic categories according to the German Commission of Hospital Hygiene and Infection Prevention, *MDR* Multidrug resistant, *UHZ-MDR* Multidrug resistance according to University Hospital Zurich guidelines

^a)^ as piperacillin/tazobactam and not piperacillin was tested: Resistance to ceftriaxone and/or ceftazidime was interpreted as resistance to Piperacillin [21]

^b)^ as cefotaxime was not tested, ceftriaxone replaces cefotaxime

^c)^ intrinsic resistance of Enterobacter claocae and *Enterobacter aerogenes*

^d)^ intrinsic resistance of Klebsiella

^e)^ only approved for *E. coli*, *K. pneumoniae* and *K. oxytoca*

*Italic font*: not tested in UHZ or only tested by MIC under special circumstances

**Table 2:** Definition criteria and antibiotic panel for *Pseudomonas aeruginosa*

Antimicrobial category	ECDC-MDR	KRINKO-3MRGN	KRINKO-4MRGN	UHZ-MDR
Antipseudomonal penicillins	Piperacillin/ tazobactam or *ticarcillin/ clavulanic acid*	*Piperacillin* ^a)^	*Piperacillin* ^a)^	Piperacillin/ tazobactam
Extended spectrum cephalosporins	Ceftazidime or cefepime	Ceftazidime and cefepime	Ceftazidime and cefepime	Ceftazidime and cefepime
Carbapenems	Imipenem or meropenem or *doripenem*	Imipenem and meropenem	Imipenem and meropenem	Imipenem or meropenem
Quinolones	Ciprofloxacin or levofloxacin	Ciprofloxacin	Ciprofloxacin	Ciprofloxacin and levofloxacin
Aminoglycosides	Amikacin or gentamicin or tobramycin or n*etilmicin*			≥2 of: Amikacin, gentamicin, tobramycin
Monobactams	*Aztreonam*			
Phosphonic acids	*Fosfomycin*			
Polymyxins	*Colistin* or *polymyxin B*			
Definition of MDR	Resistant to ≥3 categories	Resistant to 3 out of 4 categories	Resistant to all 4 categories	Resistant to 3 out of 5 categories

Abbreviations: *ECDC-MDR* Multidrug resistance according to the European Centre for Disease Prevention and Control, *KRINKO-3MRGN* Multidrug resistance to three antibiotic categories according to the German Commission of Hospital Hygiene and Infection Prevention, *KRINKO-4MRGN* Multidrug resistance to four antibiotic categories according to the German Commission of Hospital Hygiene and Infection Prevention, *MDR* multidrug resistant, *UHZ-MDR* multidrug resistance according to University Hospital Zurich guidelines

^a)^ not tested in UHZ, replaced by piperacillin/tazobactam

*Italic font*: not tested in UHZ or only tested by MIC under special circumstances

**Table 3:** Definition criteria and antibiotic panel for *Acinetobacter baumannii* complex

Antimicrobial category	ECDC-MDR	KRINKO-3MRGN	KRINKO-4MRGN	UHZ-MDR
Antipseudomonal penicillins	Piperacillin/ tazobactam or *ticarcillin/ clavulanic acid*	*Piperacillin* ^a)^	*Piperacillin* ^a)^	Piperacillin/ tazobactam
Extended spectrum cephalosporins	*Cefotaxime* or ceftriaxone or ceftazidime or cefepime	*Cefotaxime* ^b)^ or ceftazidime	*Cefotaxime* ^b)^ or ceftazidime	Ceftazidime and cefepime
Carbapenems	Imipenem or meropenem or *doripenem*	Imipenem or meropenem	Imipenem or meropenem	Imipenem or meropenem
Quinolones	Ciprofloxacin or levofloxacin	Ciprofloxacin	Ciprofloxacin	Ciprofloxacin and levofloxacin
Aminoglycosides	Amikacin or gentamicin or tobramycin or n*etilmicin*			≥2 of: Amikacin, gentamicin, tobramycin
Folate pathway inhibitors	Trimethoprim/ sulphamethoxazol			
Penicillins plus beta-lactamase-inhibitors	*Ampicillin/ sulbactam*			
Polymyxins	*Colistin* or *polymyxin B*			
Tetracyclines	Tetracycline or *doxycycline* or minocycline			
Definition of MDR	Resistant to ≥3 categories	Resistant to antipseudomonal penicillin and cephalosporin-group and quinolone and susceptible to carbapenems	Resistant to all 4 categories or If a carbapenem is resistant all Acinetobacter are interpreted as 4MRGN or Acinetobacter with carbapenemase production	Resistant to 3 out of 5 categories

Abbreviations: *ECDC-MDR* Multidrug resistance according to the European Centre for Disease Prevention and Control, *KRINKO-3MRGN* Multidrug resistance to three antibiotic categories according to the German Commission of Hospital Hygiene and Infection Prevention, *KRINKO-4MRGN* Multidrug resistance to four antibiotic categories according to the German Commission of Hospital Hygiene and Infection Prevention, *MDR* Multidrug resistant, *UHZ-MDR* multidrug resistance according to University Hospital Zurich guidelines

^a)^ as piperacillin/tazobactam and not piperacillin was tested: Resistance to ceftriaxone and/or ceftazidime was interpreted as resistance to piperacillin (15)

^b)^ as cefotaxime was not tested, ceftazidime replaces cefotaxime

*Italic font*: not tested in UHZ or only tested by MIC under special circumstances

ECDC definitions, published by Magiorakos et al. in 2011 [9]: The ECDC definitions define multidrug-resistance, ‘ECDC-MDR’, by non-susceptibility to at least one agent in three or more of 17 antimicrobial categories for Enterobacterales, of eight categories for *P. aeruginosa*, and of nine categories for *A. baumanii*.

German KRINKO definitions, published in 2012 [10]: The definition includes two MDR categories, ‘KRINKO-3MRGN’ and ‘KRINKO-4MRGN’ (MRGN = “Multiresistente gramnegative Stäbchen”, English: “multiresistant gram-negative rods”). Antibiotics and/or drug categories that are considered relevant for the MDR-definition are i) piperacillin, ii) cephalosporins (cefotaxime (or cefepime for *P.aeruginosa*), ceftazidime) iii) ciprofloxacin, and iv) carbapenems (meropenem and imipenem). ‘KRINKO-3MRGN’ and ‘KRINKO-4MRGN’ is defined by resistance to three and four categories, respectively. Carbapenemase production automatically defines ‘KRINKO-4MRGN’. Other genotypic or phenotypic test results are not considered relevant. As bacteria of both MDR categories trigger isolation precautions, we created the category KRINKO-3/4MRGN comprising species of KRINKO-3MRGN and KRINKO-4MRGN.

UHZ-definitions, developed in 2008: The UHZ-definitions classify bacteria to ‘UHZ-MDR’ by nonsusceptibility to one or several agents of at least three out of five drug categories. In addition, ESBL producing bacteria (except *E. coli*) and carbapenamase producing bacteria are always classified as ‘UHZ-MDR’, irrespective of the reported susceptibilities.

### Adaptions due to local antibiotic panel

As the Institute of Medical Microbiology of the University of Zurich tested piperacillin/tazobactam but not piperacillin alone (which is required in the KRINKO definitions), piperacillin resistance of *Enterobacterales* and *A. baumanii* complex was inferred from resistance to cefotaxime or ceftazidime as it is advised from the German KRINKO [21]. For the same reason, cefotaxime was replaced by ceftriaxone for the KRINKO definitions.

### Statistical analyses

Differences in group proportions were assessed using Fisher’s exact test. We used Stata (Version 15.1, StataCorp, College Station, Texas) for statistical analyses. In order to address incidental findings associated with multiple testing, only *P*-values <.001 were considered statistically significant.

## Results

We included 4′300 isolates of *E. coli*, 1′161 isolates of *K. pneumoniae*, 610 isolates of *Enterobacter* sp., 5′158 isolates of *P. aeruginosa* and 178 isolates of *A. baumannii* complex into the analysis. Susceptibility rates for all relevant antibiotics are shown in Additional file 1**: Figure S1**.

### Isolates classified as GN-MDRO

**Fig. 1:**
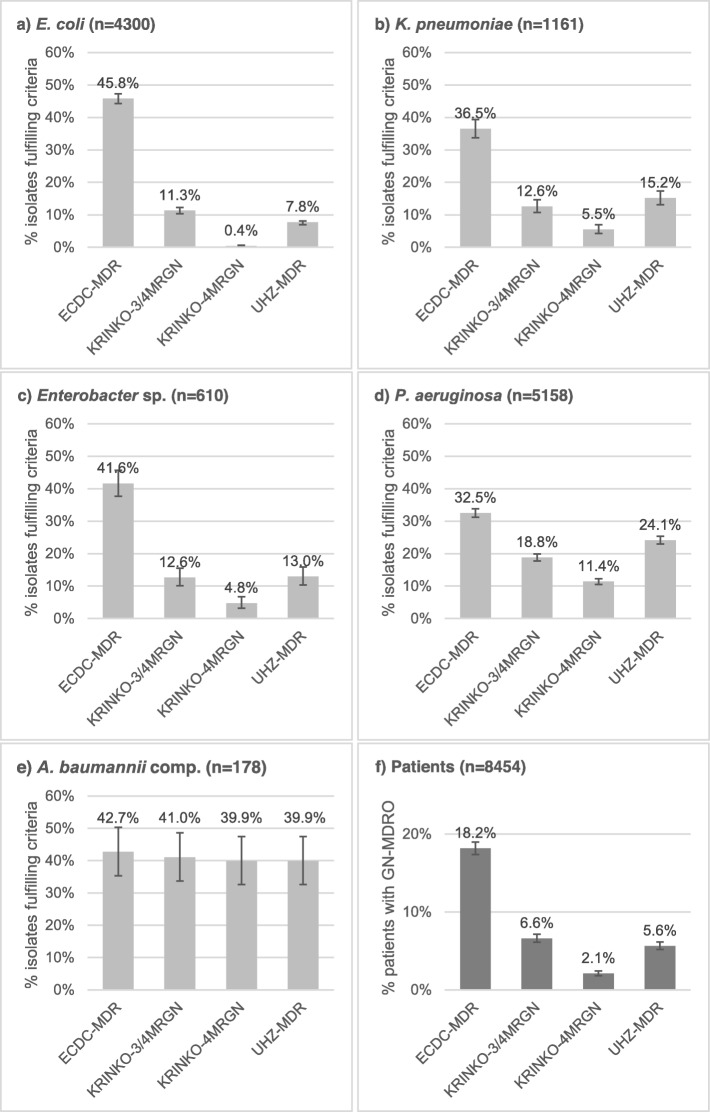
Percentages of isolates and patients fulfilling the respective GN-MDRO criteria. Light grey bars are the percentage of isolates classified as GN-MDRO according to the respective definition criteria. Grey bars are the percentage of patients who ever had an isolate fulfilling the respective definition criteria. The definition criteria are shown in Tables 1, 2 and 3. ECDC-MDR, multidrug resistance according to the European Centre for Disease Prevention and Control; GN-MDRO, Gram-negative multirdrug resistant organisms; KRINKO-3/4MRGN, multidrug resistance defined as resistance to three or four antibiotic categories according to the German Commission of Hospital Hygiene and Infection Prevention; KRINKO-4MRGN, multidrug resistance defined as resistance to four antibiotic categories according to the German Commission of Hospital Hygiene and Infection Prevention; UHZ-MDR, multidrug resistance according to University Hospital Zurich guidelines

The percentages of isolates per species fulfilling the different MDR definitions are shown in Fig. 1a-e. *E.coli, K. pneumonia, Enterobacter* sp. and *P.aeruginosa* had highest rates of multidrug resistance when analysed according to the ‘ECDC-MDR’ definitions and lowest rates when applying the ‘KRINKO-4MRGN’ definitions. Interestingly, the ‘KRINKO-3/4MRGN’ definitions did not result in uniformly lower or higher MDR rates as compared to UHZ-definitions. Among *Enterobacterales* the ‘KRINKO-3/4MRGN’ definitions caused higher rates in MDR *E. coli*, whereas in *K. pneumonia* and *Enterobacter* sp. rates were comparable. By contrast, the ‘KRINKO-3/4MRGN’ lead to a lower percentage of MDR *P. aeruginosa*. In *A. baumanii* complex all four definitions lead to comparable MDR rates. *P*-values of all comparisons are shown in the Additional file 2: Table S1.

### Patients with a GN-MDRO

The percentage of patients colonised or infected with any GN-MDRO is depicted in Fig. 1f. The percentage is highest with 18.2% (95% confidence interval (CI), 17.4–19.0%) when applying the ‘ECDC-MDR’ definition and lowest with 2.1% (95% CI, 1.8–2.4%) when applying the ‘KRINKO-4MRGN’ definition. From the definitions used to guide IPC measures, the ‘KRINKO-3/4MRGN’ definition lead to highest percentages.

## Discussion

This study demonstrated major differences in rates of GN-MDRO when applying the four definitions of the ECDC, the German KRINKO and the Swiss UHZ. Highest rates of 32–46% for *Enterobacterales*, *P. aeruginosa* and *A. baumannii* were found when applying the ‘ECDC-MDR’ definitions, which were primarily chosen to harmonize epidemiological surveillance data and are defined by acquired resistance to at least one antibiotic of any three drug categories. Lowest rates were induced by applying the ‘KRINKO-4MRGN’ definition - definitions requiring resistance in all four relevant drug categories. On the patient level, the ‘ECDC-MDR’ definitions lead to around thrice as many patients with GN-MDRO compared to the ‘UHZ-MDR’ and the ‘KRINKO-3/4MRGN’ criteria, and around nine times as many patients compared to the ‘KRINKO-4MRGN’ criteria.

MDR definitions differ between agencies, countries, and even between hospitals in a particular country [7]. A survey among members of the SHEA Research network in 2012/2013 showed that among 66 hospitals, there were 22 different MDR definitions for *Enterobacterales* [7]. The survey also revealed that the most commonly selected definition of MDR for *Enterobacterales*, *A. baumannii* complex and *P. aeruginosa* was resistance to three or more classes of antimicrobials. A literature review in 2006 showed that various definitions are used for the terms MDR *A. baumannii* complex and *P. aeruginosa* [22]. MacKinnon et al. evaluated the effects of applying three MDR classification metrics on *E. coli* isolates from chicken abattoir surveillance samples and showed that rates were highest when the isolates were non-susceptible to ≥3 antibiotic drugs (53.3%), followed by non-susceptible to ≥3 antibiotic categories (i.e. the ‘ECDC-MDR’ definition, 49.4%), and ≥ 3 antibiotic classes (38.5%) [23].

All definitions evaluated in the current study – irrespective if used for surveillance reasons or to guide IPC-precautions - use a ‘category approach’. As expected, the more categories are required to be resistant, the less isolates are considered GN-MDRO. This explains the lowest rates by applying the ‘KRINKO-4MRGN’ definition defined by resistance to all four tested categories. Regarding definition criteria requiring resistance to three drug categories (‘ECDC-MDR’, ‘KRINKO-3/4MRGN’, UHZ-definitions), differences arise from the number of categories taken into consideration, and the number of drugs in the respective drug category required to be resistant. As expected, the ‘ECDC-MDR’ definitions – with highest number of relevant drug categories (i.e. up to 17 categories compared to four or five in Enterobacterales) and lowest number of antibiotics required to be resistant per drug category (i.e. one antibiotic compared to up to three) - lead to considerably higher rates of GN-MDRO than the other two definition criteria. The difference between UHZ-MDR and KRINKO-3/4MRGN *E. coli* (7.8% vs. 11.3%) may be driven by *E.coli* ESBL producing species that fulfill the ‘KRINKO-3/4MRGN’ definition, but do not meet the UHZ-MDR definitions: Although the UHZ criteria generally consider ESBL-producing strains - detected phenotypically or genotypically - as GN-MDRO, regardless of whether the strains fulfill the ‘UHZ-MDR’ criteria otherwise, the *E. coli* ESBL isolated are treated as an exception. *E.coli* are not per se considered UHZ-MDR because *E.coli* has a demonstrated lower transmission potential than other *Enterobacterales,* and hence bear a lesser potential for outbreaks [24, 25]. In comparison, e.g. *K. pneumoniae* shows a clear trend to spread clonally within healthcare institutions, and *K. pneumoniae* ESBL are considered UZH-MDR irrespective ot other drug resistances being presented or not [26].

When applying the three definition criteria used to guide IPC measures (both KRINKO and the UHZ definition) on the patient level, only 2% of patients are colonized or infected with bacteria fulfilling the ‘KRINKO-4MRGN’ definitions. The UHZ-definition and the ‘KRINKO-3/4MRGN’ definition lead to 5.6 and 6.6% of patients with an GN-MDRO, respectively. The difference between the UHZ-definitions and the ‘KRINKO-3/4MRGN’ definition is driven by the difference in MDR *E. coli*, the only species in which UHZ-definitions showed lower results than KRINKO-3/4MRGN. This relative difference of 16% between UHZ- and ‘KRINKO-3/4MRGN’ definitions still is considerable when taking into account that most hospitals would assign patients with GN-MDRO to a single room and ask for resource intensive isolation precautions. A study by Tran et al. showed that patients isolated for methicillin resistant *Staphylococcus aureus* in comparison to non-isolated patients had longer lengths of hospital stay, stayed in hospital longer than expected, and had higher costs [5]. Direct costs of isolation precautions in a Swiss hospital were calculated to sum up to 158 $ per day [4].

Our study has limitations. First, it was conducted in a low prevalence country for GN-MDRO and our results might not be similarly applicable to higher prevalence regions. Second, the antibiotic panel tested has a critical influence on resistance rates, especially for the ‘ECDC-MDR” definition. Broadly speaking, the higher the number of antibiotics on the panel the higher the probability that an isolate is considered ‘ECDC-MDR’. The ECDC emphazises the necessity of testing an adequate number of antimicrobial agents in order to effectively apply the ECDC-definitions, but even in a high-income country like Switzerland, the antibiotic panel tested for *Enterobacterales* is smaller than the proposed ECDC panel. This is due to financial restrictions that force clinical laboratories to work at least cost-neutral. Also, manufacturers of microdilution assays often preset the antibiotic panel tested. These facts extrinsicaly cause differences in ‘ECDC-MDR’ rates and preclude comparisons between hospitals with different antibiotic panel. Still, as we were unable to identify other studies trying to answer this research question, our study provides unique data demonstrating the difference of GN-MDRO rates after applying diverse resistance definitions to a “real-world” clinical database with a “real-world” antibiotic panel.

## Conclusion

By demonstrating that rates of gram-negatives classified as MDRO differ considerably depending on the applied MDR-criteria, we consider choosing definition criteria carefully depending on their purpose to be of high importance. For determining definitions directing isolation precautions, local resistance rates and epidemiological priorities as well as available resources should be taken into consideration. The additional effort and costs of isolation precautions should be in due proportion to the expected benefit. GN-MDRO abundantly present in the population likely do not require isolation precautions. MDR definitions should be consistent, clearly communicated, and reviewed regularly. More studies applying the different definitions on datasets from high prevalence regions or testing the effects of other definitions would provide useful information to further get insight in the landscape of GN-MDRO worldwide. Additionally, the effect of EUCASTs new definitions of susceptibility testing categories would be interesting to evaluate [27].

## Supplementary information

**Additional file 1: Figure S1.** Susceptibility rates of Escherichia coli, Klebsiella pneumoniae, Enterobacter sp., Pseudomonas aeruginosa and Acinetobacter baumannii complex to the tested antibiotics.

**Additional file 2: Table S1.** Cross tabulations of MDR rates and p-values.

## Data Availability

The datasets used and/or analysed during the current study are available from the corresponding author on reasonable request.

## Abbreviations

ECDC-MDR: Multidrug resistance according to ECDC definitions
MDR: Multidrug resistance
ECDC: European Centre for Disease Prevention and Control
GN-MDRO: Gram-negative multidrug-resistant organisms
IPC: Infection prevention and control
KRINKO: German Commission of Hospital Hygiene and Infection Prevention (German: Kommission für Krankenhaushygiene und Infektionsprävention)
KRINKO-3/4MRGN: Multidrug resistance to three or four drug categories according to KRINKO definitions
KRINKO-3MRGN: Multidrug resistance to three drug categories according to KRINKO definitions
KRINKO-4MRGN: Multidrug resistance to four drug categories according to KRINKO definitions
MDR: Multidrug resistance
UHZ: University Hospital Zurich
UHZ-MDR: Multidrug resistance according to the University Hospital Zurich guidelines
