# Supplementary material for: The effect of varying multidrug-resistence (MDR) definitions on rates of MDR gram-negative rods
Source: Antimicrob Resist Infect Control. 2019 Nov 28;8:193. doi: 10.1186/s13756-019-0614-3 (PMC6883537; doi:10.1186/s13756-019-0614-3)
Supplement: Supplementary file 1 — Additional file 1: Figure S1. Susceptibility rates of Escherichia coli, Klebsiella pneumoniae, Enterobacter sp., Pseudomonas aeruginosa and Acinetobacter baumannii complex to the tested antibiotics. [file 13756_2019_614_MOESM1_ESM.pdf]

Supplement Figure 1 - Susceptibility rates of *Escherichia coli*, *Klebsiella pneumoniae*, *Enterobacter* sp., *Pseudomonas aeruginosa* and *Acinetobacter baumannii* complex to the tested antibiotics

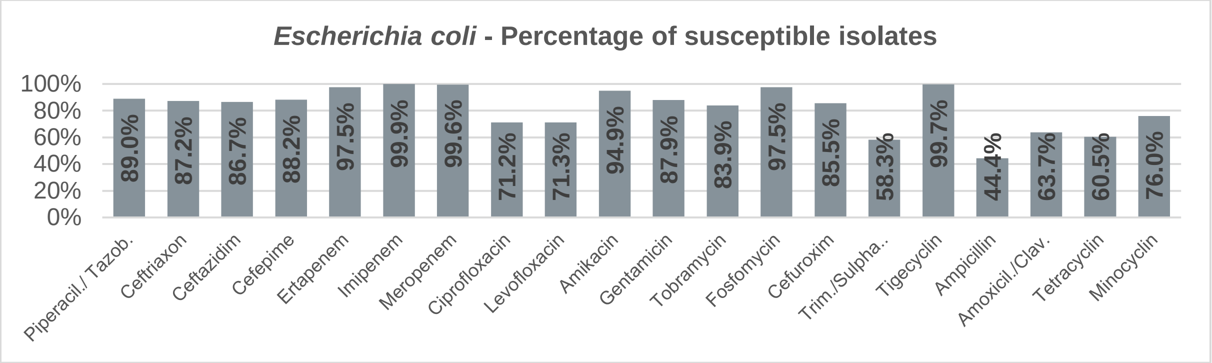

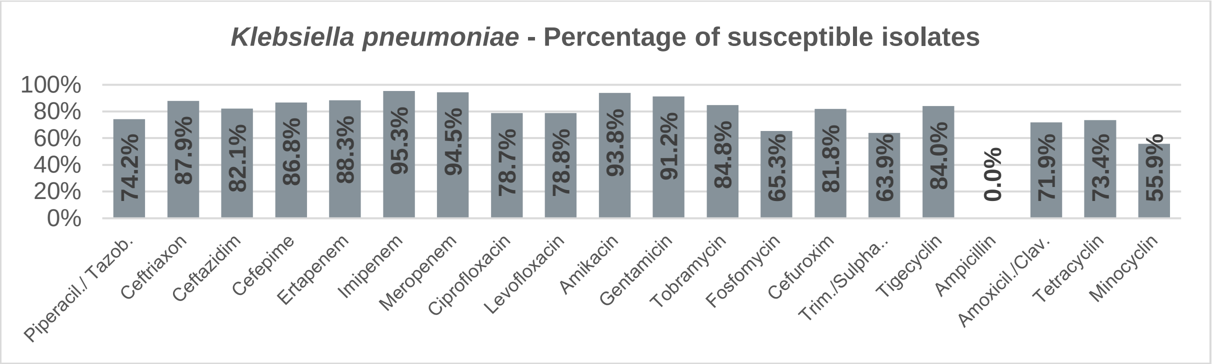

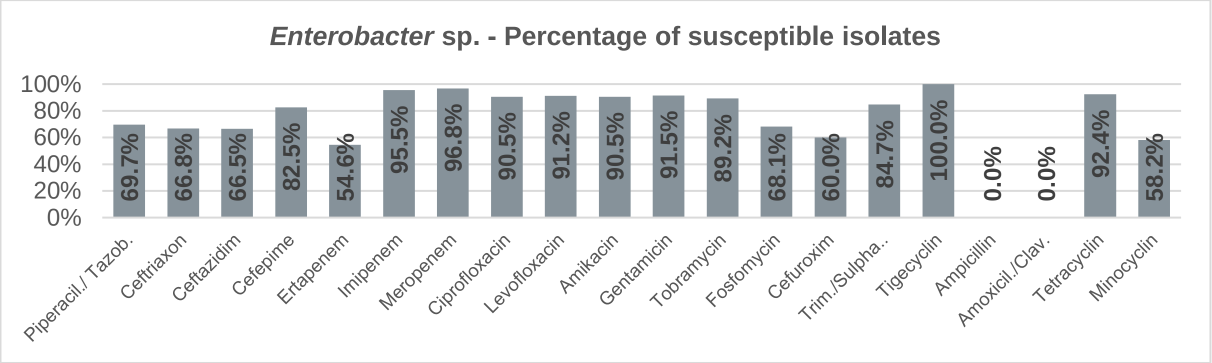

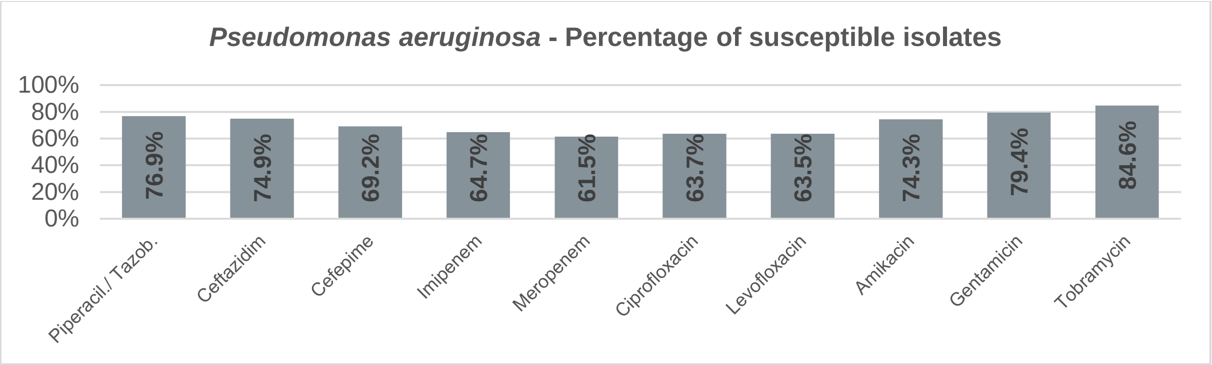

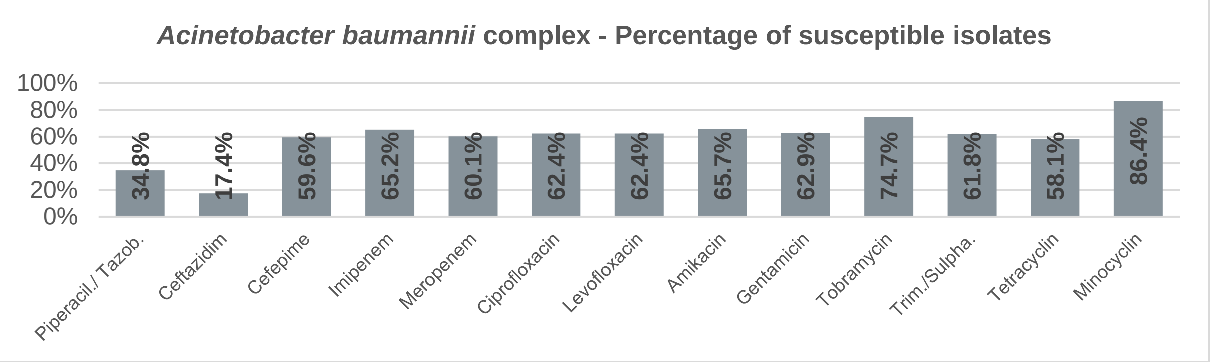

Acinetobacter baumannii complex - Percentage of susceptible isolates
100%
80%
60%
40%
20%
0%
34.8%
17.4%
59.6%
65.2%
60.1%
62.4%
62.4%
65.7%
62.9%
74.7%
61.8%
58.1%
86.4%
Piperacil./ Tazob.
Ceftazidim
Cefepime
Imipenem
Meropenem
Ciprofloxacin
Levofloxacin
Amikacin
Gentamicin
Tobramycin
Trim./Sulpha.
Tetracyclin
Minocyclin
