# Supplementary material for: The effect of varying multidrug-resistence (MDR) definitions on rates of MDR gram-negative rods
Source: Antimicrob Resist Infect Control. 2019 Nov 28;8:193. doi: 10.1186/s13756-019-0614-3 (PMC6883537; doi:10.1186/s13756-019-0614-3)
Supplement: Supplementary file 2 — Additional file 2: Table S1. Cross tabulations of MDR rates and p-values. [file 13756_2019_614_MOESM2_ESM.docx]

**Supplement Table: Cross tabulations of MDR rates and p-values**

***E.coli* – Cross tabulation with p-values**

| Percentage MDR |  | 45.8% | 11.3% | 0.4% | 7.8% |
| --- | --- | --- | --- | --- | --- |
|  | **Definition criteria** | **ECDC-MDR** | **KRINKO-3/4MRGN** | **KRINKO-4MRGN** | **UHZ-MDR** |
| 45.8% | **ECDC-MDR** | **n.a.** | **0.000** | **0.000** | **0.000** |
| 11.3% | **KRINKO-3MRGN** | **0.000** | **n.a.** | **0.000** | **0.000** |
| 0.4% | **KRINKO-4MRGN** | **0.000** | **0.000** | **n.a.** | **0.000** |
| 7.8% | **UHZ-MDR** | **0.000** | **0.000** | **0.000** | **n.a.** |

***K. pneumoniae*– Cross tabulation with p-values**

| Percentage MDR |  | 36.5% | 12.6% | 5.5% | 15.2% |
| --- | --- | --- | --- | --- | --- |
|  | **Definition criteria** | **ECDC-MDR** | **KRINKO-3/4MRGN** | **KRINKO-4MRGN** | **UHZ-MDR** |
| 36.5% | **ECDC-MDR** | **n.a.** | **0.000** | **0.000** | **0.000** |
| 12.6% | **KRINKO-3MRGN** | **0.000** | **n.a.** | **0.000** | **0.082** |
| 5.5% | **KRINKO-4MRGN** | **0.000** | **0.000** | **n.a.** | **0.000** |
| 15.2% | **UHZ-MDR** | **0.000** | **0.082** | **0.000** | **n.a.** |

***Enterobacter* sp. – Cross tabulation with p-values**

| Percentage MDR |  | 41.6% | 12.6% | 4.8% | 13.0% |
| --- | --- | --- | --- | --- | --- |
|  | **Definition criteria** | **ECDC-MDR** | **KRINKO-3/4MRGN** | **KRINKO-4MRGN** | **UHZ-MDR** |
| 41.6% | **ECDC-MDR** | **n.a.** | **0.000** | **0.000** | **0.000** |
| 12.6% | **KRINKO-3MRGN** | **0.000** | **n.a.** | **0.000** | **0.932** |
| 4.8% | **KRINKO-4MRGN** | **0.000** | **0.000** | **n.a.** | **0.000** |
| 13.0% | **UHZ-MDR** | **0.000** | **0.932** | **0.000** | **n.a.** |

***P. aeruginosa* – Cross tabulation with p-values**

| Percentage MDR |  | 32.5% | 18.8% | 11.4% | 24.1% |
| --- | --- | --- | --- | --- | --- |
|  | **Definition criteria** | **ECDC-MDR** | **KRINKO-3/4MRGN** | **KRINKO-4MRGN** | **UHZ-MDR** |
| 32.5% | **ECDC-MDR** | **n.a.** | **0.000** | **0.000** | **0.000** |
| 18.8% | **KRINKO-3MRGN** | **0.000** | **n.a.** | **0.000** | **0.000** |
| 11.4% | **KRINKO-4MRGN** | **0.000** | **0.000** | **n.a.** | **0.000** |
| 24.1% | **UHZ-MDR** | **0.000** | **0.000** | **0.000** | **n.a.** |

***Acinetobacter*** ***baumannii*** **complex – Cross tabulation with p-values**

| Percentage MDR |  | 42.7% | 41.0% | 39.9% | 39.9% |
| --- | --- | --- | --- | --- | --- |
|  | **Definition criteria** | **ECDC-MDR** | **KRINKO-3/4MRGN** | **KRINKO-4MRGN** | **UHZ-MDR** |
| 42.7% | **ECDC-MDR** | **n.a.** | **0.830** | **0.667** | **0.667** |
| 41.0% | **KRINKO-3MRGN** | **0.830** | **n.a.** | **0.914** | **0.914** |
| 39.9% | **KRINKO-4MRGN** | **0.667** | **0.914** | **n.a.** | **1.000** |
| 39.9% | **UHZ-2008** | **0.667** | **0.914** | **1.000** | **n.a.** |

**Patients with MDR colonization/infection – Cross tabulation with p-values**

| Percentage MDR |  | 18.2% | 6.6% | 2.1% | 5.6% |
| --- | --- | --- | --- | --- | --- |
|  | **Definition criteria** | **ECDC-MDR** | **KRINKO-3/4MRGN** | **KRINKO-4MRGN** | **UHZ-MDR** |
| 18.2% | **ECDC-MDR** | **n.a.** | **0.000** | **0.000** | **0.000** |
| 6.6% | **KRINKO-3MRGN** | **0.000** | **n.a.** | **0.000** | **0.007** |
| 2.1% | **KRINKO-4MRGN** | **0.000** | **0.000** | **n.a.** | **0.000** |
| 5.6% | **UHZ-2008** | **0.000** | **0.007** | **0.000** | **n.a.** |

Dark grey: Percentage of isolates fulfilling the respective MDR definition. Light gray: Definition criteria of MDR definitions. White: p values. ECDC-MDR, multidrug resistance according to the European Centre for Disease Prevention and Control; KRINKO-4MRGN, multidrug resistance defined as resistance to four antibiotic categories according to the German Commission of Hospital Hygiene and Infection Prevention; MDR, multirdrug resistance; UHZ-MDR, multidrug resistance according to University Hospital Zurich guidelines.
